# Sustaining and Expanding Internet-Delivered Cognitive Behavioral Therapy (ICBT) for Public Safety Personnel across Canada: A Survey of Stakeholder Perspectives

**DOI:** 10.3390/ijerph20085592

**Published:** 2023-04-20

**Authors:** Caeleigh A. Landry, Janine D. Beahm, Hugh C. McCall, Heather D. Hadjistavropoulos

**Affiliations:** 1 Department of Psychology, University of Regina, 3737 Wascana Pkwy, Regina, SK S4S 0A2, Canada; 2 PSPNET, University of Regina, 2 Research Drive, Regina, SK S4T 2P7, Canada

**Keywords:** internet-delivered cognitive behavioral therapy, public safety personnel, first responders, implementation research, program sustainment, occupational injuries

## Abstract

Public safety personnel (PSP) experience an elevated risk of mental health problems and face barriers to treatment. Internet-delivered cognitive behavioral therapy (ICBT) has been tailored to PSP to improve access to mental health care. In this study, we sought to investigate perceptions of ICBT, particularly among those with and without prior knowledge of ICBT and between PSP leaders and non-leaders. A survey was administered to 524 PSP from across Canada to identify (a) how PSP perceive ICBT, (b) the extent of organizational support for tailored ICBT in PSP organizations, particularly leadership’s support, and (c) perceived facilitators and barriers to funding tailored ICBT. The results indicated that PSP perceive ICBT to have more advantages than disadvantages. PSP who had previously heard of tailored ICBT had more positive perceptions. PSP indicated that there is a need for ICBT, and PSP leaders indicated their support for the implementation of tailored ICBT. The study identified that there is a need for increasing awareness of the effectiveness of and need for ICBT in order to facilitate funding of services. Overall, the current study indicates that PSP support ICBT as a valued form of therapy and that policy makers and service providers seeking to provide ICBT to PSP may increase support for ICBT services through more education and awareness.

## 1. Introduction

The umbrella term public safety personnel (PSP) refers to a diverse set of vocations dedicated to ensuring the safety and wellbeing of communities. PSP include, but are not limited to, border services officers, correctional workers, fire fighters (career and volunteer), Indigenous emergency managers, operational intelligence personnel, paramedics, police (municipal, provincial, and federal), public safety communicators, and search and rescue personnel [1]. PSP are routinely exposed to potentially psychologically traumatic events (e.g., violence, explosions), which have been associated with an increased risk of developing post-traumatic stress injuries (PTSIs) [2,3]. The Canadian House of Commons Standing Committee on Public Safety and National Security identified PTSIs experienced by PSP while serving as a matter of growing concern [4]. The Committee highlighted the need for a national strategy on PTSIs that would include evidence-based programs to facilitate education, prevention, screening, and treatment of PTSIs among PSP. To support the need for such interventions and to investigate the severity and frequency of mental disorder symptoms among Canadian PSP, a pan-Canadian study including 5813 PSP across a broad range of sectors was conducted, finding that 44.5% of respondents reported clinically significant symptoms of one or more mental disorders on questionnaire measures [5].

In response to these findings, in 2019, the Government of Canada announced a CAD 10 million investment over five years to develop and pilot an internet-delivered cognitive behavioral therapy (ICBT) program tailored to PSP in order to improve access to treatment for PSP [6]. As a result, PSPNET, a clinical research unit, was established to offer and evaluate ICBT tailored to meet the needs of PSP in Canada. ICBT refers to a form of psychotherapy that retains aspects and concepts of traditional, face-to-face cognitive behavioral therapy but is delivered online, typically through a series of modules and often with therapist support by email or phone [7]. ICBT was chosen because it has been shown to be effective for treating symptoms of common mental disorders (e.g., anxiety, depression, posttraumatic stress) [7,8,9,10] and because it is accessible and can overcome barriers to mental health care, including many of the logistical and attitudinal barriers to treatment that PSP report [11,12,13]. It has also been shown to be cost-effective [14].

Rather than developing a new ICBT program, PSPNET tailored an existing ICBT course, called the Wellbeing Course, for PSP. This course, which is transdiagnostic (i.e., designed to treat symptoms of multiple mental disorders) and therapist-guided, has already been shown to be effective among the general population in Australia and the Canadian province of Saskatchewan [15,16,17,18,19,20,21,22,23]. PSPNET carried out two studies to explore Canadian PSP’s perspectives on ICBT and to help tailor the Wellbeing Course for them [13,24]. In these studies, PSP from Saskatchewan and Quebec identified both advantages and disadvantages of ICBT. PSP, including both frontline personnel and leaders, also reported perceiving a need for ICBT, reported having favorable perceptions of ICBT, and offered input on how to tailor ICBT to PSP [13,24].

The tailored version of the Wellbeing Course—titled the PSP Wellbeing Course—was implemented in Saskatchewan and Quebec. The PSP Wellbeing Course contains five core lessons and weekly therapist guidance by phone or by email. More details on the course can be found elsewhere [25]. Initial results from clients enrolled within the first six months in Saskatchewan suggest that the PSP Wellbeing Course is effective for treating symptoms of depression, anxiety, PTSD, panic disorder, and anger among PSP [25] and is perceived as beneficial by the majority of clients [26]. While the above results were encouraging, they were conducted before or early in the process of implementing PSPNET. There remains a need to understand perceptions of ICBT after a longer period of implementation, as it is unknown if perceptions remained the same after implementation. This is particularly important because there is an increasing emphasis on using digital mental health interventions, such as ICBT, within PSP populations [27,28,29], but there is still limited evidence on the perceptions of these interventions within PSP populations.

There are mixed findings in non-PSP populations regarding the acceptance of ICBT and digital mental health interventions in various countries, with some studies showing significant acceptance of digital mental health interventions and ICBT and others showing negative attitudes [30,31,32,33,34]. There is some evidence that perceptions of an increased need for mental health treatments, limited access to services, and lower computer anxiety are related to increased acceptability of ICBT [35]. Moreover, there is evidence in non-PSP populations that increased knowledge of ICBT is related to more positive perceptions of ICBT [36,37], but the degree to which this may be true of PSP currently remains unknown. Understanding the relationship between knowledge and perceptions of ICBT among PSP can inform whether education on ICBT is likely to facilitate efforts to sustain and expand ICBT. Overall, understanding PSP’s perceptions of the advantages or disadvantages of ICBT is important because positive perceptions of innovation characteristics are found to be essential for successful implementation [38,39,40].

Past research on digital mental health interventions also emphasizes the importance of leadership support and engagement at all stages of development and implementation [38,39,41]. Therefore, it is important to gauge PSP leaders’ perceptions of ICBT. Finally, sufficient financial support needs to be in place to ensure the successful implementation of digital mental health interventions [39]. Understanding PSP’s perceptions of options for funding and factors that may facilitate or hinder funding can provide valuable insights into funding opportunities for ICBT among PSP.

Therefore, the current study aimed to expand on previous PSPNET stakeholder research and address the following exploratory questions:

1. To what extent do PSP perceive a need for ICBT, and does this differ between stakeholders with prior knowledge of PSPNET and those without, as well as between leaders and non-leaders?
2. What advantages and concerns do PSP identify about ICBT, and do perceptions differ between stakeholders with prior knowledge of PSPNET and those without, as well as between leaders and non-leaders?
3. What are PSP leaders’ perspectives on how PSPNET should be implemented? Specifically, how do PSP leaders believe that their organization can support PSPNET, how do they believe PSPNET should be funded, and what factors do they perceive as facilitators or impediments to funding PSPNET?

## 2. Materials and Methods

### 2.1. Sample and Setting

Five hundred and fifty-seven participants began the survey. Thirty-three participants were excluded from the analyses because they answered fewer than 78% of the questions or failed to answer key questions that were used to group participants in the analyses. A series of *t*-tests and chi-squares were conducted and showed there were no statistically significant differences between those included and excluded based on the available demographic information. The final sample included 524 PSP from across Canada. The mean age of participants was 43 years old (*SD* = 10.8). The sample primarily self-identified as white (85.4%), and a slight majority self-identified as male (53.2%). PSP were recruited from several sectors, including police (24.8%), paramedics (17.7%), correctional workers (23.3%), firefighters (12.7%), and public safety communicators (9.1%). Half of the participants (49.9%) reported they were in a leadership role within their organization. Most participants (72%; *n* = 377) reported they had not heard of PSPNET prior to being invited to participate in the survey. Most also reported that they had not previously used PSPNET (97%) or any form of online therapy (93%; *n* = 504). Of note, PSPNET was only providing services in Saskatchewan and Quebec at the time the present study was launched and began providing services in Prince Edward Island and Nova Scotia during the study. Details can be found in Table 1.

### 2.2. Materials and Data Collection

Participants for the study were recruited through social media and snowball sampling. Participants were sent an email inviting them to participate in an online survey. Interested participants were invited to follow a link to an informed consent form. After consenting to participate, participants were asked to complete demographic questions, view a short video about PSPNET and a brochure about PSPNET services (https://youtu.be/lzhrMNRv-Ac; see Supplementary Material S1 for the transcript and brochure), and then answer an online bespoke questionnaire concerning their perceptions of PSPNET and ICBT. The questionnaire included both closed and open-ended questions. The 36 closed-ended questions included four subsections of questions and addressed perceptions of the characteristics of and support for PSPNET and ICBT in the organizational setting. In the first set of questions, participants were presented with several potential advantages of the innovation characteristics of tailored ICBT or PSPNET that were identified by participants in previous studies [13,24]; they were then asked to rate each on a 4-point Likert scale ranging from 1 (not an advantage) to 4 (major advantage). In the second set of questions, participants were asked about their concerns regarding the characteristics of tailored ICBT or PSPNET and to rate their responses on a 4-point Likert-type scale ranging from 1 (not a concern) to 4 (major concern). In the third set of questions, participants were asked about their perceptions of their organizational setting concerning the mental health challenges of PSP and the potential usefulness of PSPNET to respond to those challenges and provided responses on a 5-point Likert-type scale ranging from 1 (strongly disagree) to 5 (strongly agree). PSP who identified as leaders were asked to complete a fourth set of questions concerning their perceptions of a need for ICBT and whether they believed their organization would support ICBT. Participants (i.e., self-identified leaders) rated their responses on a 5-point Likert-type scale ranging from 1 (strongly disagree) to 5 (strongly agree). There were three open-ended questions, which were used to explore participants’ perceptions of how PSPNET ought to be funded and what factors might facilitate or impede funding of PSPNET. The survey was designed to take approximately 20–25 min to complete. The survey was administered between November 2021 and March 2022. This study was approved by the University of Regina Research Ethics Board (REB #2021-144).

### 2.3. Data Analysis

Data from the closed-ended survey questions were imported into IBM SPSS Statistics v.26 [42], and descriptive statistics were computed. Next, four one-way analyses of variance (ANOVAs) were conducted in order to determine whether there were any differences in ratings of the characteristics and perceptions of the need for PSPNET between those who had and had not heard of PSPNET, as well as between those who did and did not self-identify as leaders. A fifth ANOVA was conducted to assess leader-specific attitudes between leaders who had and had not heard of PSPNET. Data from the open-ended responses were de-identified and uploaded into Nvivo 20 [43]. The qualitative responses were analyzed using a conventional qualitative content analysis [44]. Author C.A.L. and a research associate first familiarized themselves with the data. The data were initially coded by a research associate, who established categories by identifying units of meaning expressed by two or more participants. Author C.A.L. and the research associate then met to discuss the coding of categories and resolve disagreements. Data were re-coded during this review to ensure the data were coded consistently.

## 3. Results

### 3.1. Summary of Quantitative Data

#### 3.1.1. Perceptions of Need for Mental Health and ICBT Services

Overall, participants reported agreeing and strongly agreeing that mental health problems are common in their occupation and that access to PSPNET may be beneficial. See Table 2 for details.

Participants who had previously heard of PSPNET were more likely to report that PSPNET could help people in their occupations improve their mental health, *F*(1,521) = 8.32, *p* = 0.004, that PSPNET should be easily and freely accessible to people in their organization, *F*(1,521) = 3.98, *p* = 0.047, and that they have experienced mental health concerns for which they have had difficulty accessing professional help, *F*(1,520) = 7.86, *p* = 0.005. Participants who had not previously heard of PSPNET were more likely to indicate a preference to use some other service, *F*(1,508) = 11.36, *p* < 0.001. No differences were found between self-identified PSP leaders and non-leaders.

#### 3.1.2. Perceptions of Advantages of ICBT

Most respondents reported positive perceptions of PSPNET’s characteristics. See Table 3 for a summary of the findings.

Results of the ANOVAs showed that some responses differed based on whether participants had previously heard of PSPNET and whether they self-identified as a leader within their organization. On average, those who had previously heard of PSPNET rated several characteristics of ICBT services as more advantageous: that services begin with an initial online and telephone assessment to determine fit, *F*(1,522) = 3.86, *p* = 0.05; that therapist guidance is available for up to 16 weeks, *F*(1,520) = 6.84, *p* = 0.009; that a referral for other services can be made if necessary, *F*(1,522) = 4.57, *p* = 0.033; that there are many stories and examples from PSP, *F*(1,519) = 6.12, *p* = 0.014; that PSPNET services are tailored for PSP, *F*(1,519) = 5.65, *p* = 0.018; that it has online delivery of information that includes easy-to-read text, audio, and video, *F*(1,519) = 6.40, *p* = 0.01; that it has greater privacy than other treatments, *F*(1,521) = 4.80, *p* = 0.029; and that PSPNET complements existing services, *F*(1,519) = 6.41, *p* = 0.012. Leaders rated two characteristics as more advantageous than non-leaders; namely, that the website is easy to use, *F*(1,521) = 5.83, *p* = 0.016, and the online delivery of information that includes easy-to-read text, audio, and video, *F*(1,519) = 6.16, *p* = 0.013.

#### 3.1.3. Negative Perceptions or Concerns about ICBT

Participants ratings of concerns related to the characteristics of tailored ICBT are shown in Table 4.

PSP who had not previously heard of PSPNET reported greater concern about the confidentiality of information than PSP who had previously heard of PSPNET, *F*(1,522) = 5.22, *p* = 0.023. There were no differences between leaders and non-leaders in the endorsement of any concerns about PSPNET.

#### 3.1.4. Leaders’ Perceptions and Support of ICBT Services

Of the 262 participants who self-identified as having a leadership role, 255 (97.3%) completed an additional survey. Overall, self-identified leaders reported believing that addressing mental health challenges within the organization is a priority and that PSPNET may be able to help address that need. See Table 5 for details.

Some leaders’ perspectives differed based on whether they had previously heard of PSPNET. Leaders who previously heard of PSPNET were more likely to believe that having PSPNET available within their organization should be a priority, *F*(1,253) = 7.55, *p* = 0.006, that PSPNET would be effective for improving the mental health of members within their organization, *F*(1,252) = 6.08, *p* = 0.014, that if PSPNET were available to people in their organization, their organization would be able to easily promote it, *F*(1,251) = 5.20, *p* = 0.023, and that they would personally advocate for people in their organization to have PSPNET, *F*(1,205) = 11.43, *p* < 0.001.

### 3.2. Summary of Qualitative Data

#### 3.2.1. Funding Recommendations

Overall, 197 of the 262 self-identified leaders (75%) had one or more responses to the open-ended section of the questionnaire. Of the 197 participants, 153 (78%) responded to the question, “What do you think is the best way to fund PSPNET so it can continue and be expanded to other provinces?” Table 6 highlights the major categories identified in participants’ responses to this question.

#### 3.2.2. Perceptions of Factors That Will Facilitate Funding of PSPNET

Of the 197 participants who provided one or more responses, 101 (51%) responded to the question, “What factors do you believe could help PSPNET obtain ongoing funding to support PSP in your province?” Table 7 highlights the major categories identified in participants’ responses with the frequency of responses.

#### 3.2.3. Perceptions of Barriers for Obtaining New Funding for Sustaining PSPNET

Of the 197 participants who provided one or more responses, 123 (62%) responded to the question, “What factors do you believe will be barriers to PSPNET obtaining ongoing funding in the future to support PSP in your province?” Table 8 highlights the categories identified in participants’ responses.

## 4. Discussion

ICBT has recently been tailored for and offered to PSP, demonstrating promising initial outcomes [25]. The current study involved a survey administered to PSP across Canada approximately 2 years after implementing ICBT for PSP in Saskatchewan and 1.5 years after implementing ICBT for PSP in Quebec. It was designed to contribute to the research literature exploring PSP’s perceptions of tailored ICBT and, specifically, the extent to which perceptions may differ among those familiar and unfamiliar with ICBT and among PSP in leadership and non-leadership positions. Moreover, as leaders are uniquely positioned to influence services available to PSP [45], we sought to better understand perceptions of ICBT among PSP leaders. Lastly, the current study was designed to identify ideas for obtaining new funding for sustaining ICBT, as well as perceptions of factors that may facilitate funding or be barriers to funding for ICBT tailored to PSP.

### 4.1. Perceptions of Characteristics

On average, participants’ endorsements of the potential advantages of ICBT as advantageous were stronger than their endorsements of potential concerns, suggesting generally favorable attitudes toward ICBT. Every potential advantage listed was perceived, on average, as being a moderate to major advantage. This suggests that after two years of implementing ICBT for PSP, perceptions of ICBT remain positive. This finding aligns with previous PSPNET stakeholder studies showing positive perceptions of ICBT among PSP in Saskatchewan and Quebec [13,24]. It contributes to a broader literature showing mixed attitudes towards digital mental health interventions. For example, recent surveys of attitudes toward digital mental health interventions include a large Canadian study in which 51% of participants reported an interest in having access to digital mental health interventions [31]; two German studies showing poor acceptance of digital mental health interventions [30,32]; a German study showing that 66% of participants perceived internet interventions as useful or helpful [33]; and a British study showing negative attitudes towards computerized mental health treatments, despite a recognition of their convenience [34]. There is also some evidence that attitudes towards digital mental healthcare services improved during the COVID-19 pandemic; for instance, a recent South Korean study found that 35% of participants reported hesitance to attend an offline clinic due to fear of contracting COVID-19, and 54% reported a preference for digital therapeutics [46]. The present findings and those of two prior studies [13,24] suggest that Canadian PSP tend to have more favorable attitudes towards ICBT than past research participants from around the world have had towards digital mental health interventions. Possible reasons for this include PSPNET’s tailoring of ICBT to meet Canadian PSP’s unique needs and PSPNET’s use of an engaging video to introduce its services to PSP. Other possible reasons include PSP’s high perceived need for mental health services, limited options for other services that meet their needs, and high computer literacy within PSP populations [13,35].

The results regarding the perceived advantages of ICBT align with factors that facilitate the implementation of digital mental health interventions according to the “innovation domain” of the Consolidated Framework for Implementation Research (CFIR) [38,39,40]. These results are promising because the CFIR is a validated framework that provides insight into whether and why various program implementation methods work [38,40]. Participants also endorsed concerns about some aspects of ICBT. However, none of the listed disadvantages were rated, on average, as a moderate or major disadvantage. The concerns about ICBT also aligned with constructs on the innovation domain that can lead to the unsuccessful implementation of digital mental health interventions, according to the CFIR [38,39,40]. For an overview of how the individual survey items align with constructs in the innovation domain of the CFIR, see Supplementary Table S1. Overall, the results support the idea that implementation of ICBT in PSP populations is likely to be successful, given that the advantages of ICBT were endorsed more strongly than concerns and because the advantages align with constructs that facilitate successful implementation [38,40].

When comparing the perceived advantages of the characteristics of PSPNET and ICBT between PSP who had previously heard of PSPNET and those who had not previously heard of PSPNET, PSP who had previously heard of PSPNET were significantly more likely to endorse several advantages more strongly than those who had not heard of PSPNET. This suggests that more awareness about ICBT is helpful for garnering support for ICBT as a new service for PSP. Previous research supports this idea and has shown that education on ICBT can increase interest in and the perceived credibility of ICBT in non-PSP populations [36,37]. Concerns remained relatively consistent among those who had heard and not heard about PSPNET; those who had not heard of PSPNET only reported greater concern regarding one innovation characteristic (i.e., concerns about the confidentiality of information). The current findings on concerns echo the findings of previous PSPNET stakeholder studies [13,24], suggesting that two years after implementation, there are still similar concerns about ICBT that need to be managed.

### 4.2. Support for ICBT in the Organizational Setting

The findings of the current study strongly suggest that PSP are aware of the need for mental health services and that PSP stakeholders believe there is a need for ICBT services. PSP leaders are supportive of ICBT and believe there is a need for PSPNET services within their organization. The results are promising for service providers seeking to implement digital mental health services for PSP. Previous implementation research suggests that organizations that have supportive attitudes towards an intervention or program, particularly leaders as well as enthusiastic and motivated champions with supportive attitudes, are facilitating factors for successful implementation and sustainability [38,39,40,47]. Research on programs developed for PSP also suggests programs are more likely to be successful when they have support from leadership [45,48]. The results also showed that PSP who have heard of PSPNET, including both leaders and non-leaders, were more likely to endorse a need for ICBT services. This also highlights the importance of continued education on ICBT to increase acceptability [36,37], particularly among PSP leaders who have a strong influence over implementation efforts [39,45,47,48].

### 4.3. Facilitators and Barriers to Funding PSPNET

Successful implementation of any intervention requires sufficient and adequate funding [39]. In order to sustain ICBT services for PSP, identifying sources of funding is required, and PSP leaders can offer insights into how to best fund services to meet their needs. Participants largely reported believing that PSPNET should be funded, specifically through provincial and federal governments, to help ensure large-scale access to its services. Despite believing the government should fund PSPNET, participants reported believing that the government does not prioritize the needs of PSP and that there is a lack of understanding of the work that PSP do. In order to facilitate funding, the results suggest that PSPNET needs to continue to engage in outreach and media strategies to PSP, PSP executives, the public, and those in government. The strategy should use targeted content that addresses PSP mental health issues, highlights PSPNET’s track record of success, and emphasizes the important role tailored ICBT services play as a cost-effective alternative to in-person therapy. This type of outreach strategy may increase political support in the external environment, which can increase provisions of funding [41].

### 4.4. Implications

The results from the study are intended to help researchers, clinicians, policy makers, PSP, and other stakeholders develop a deeper understanding of the current need for ICBT services among PSP, as well as the barriers and facilitating factors impacting the demand and the sustainability of funding for ICBT. This understanding can inform efforts to implement and sustain ICBT services for PSP. For instance, the results may provide promise to other service providers by showing that PSP, and particularly PSP leaders, believe ICBT is valuable and needed and are willing to support its implementation. Moreover, the results indicate that increasing education and awareness of ICBT can facilitate positive perceptions of it. The results also show which innovation characteristics of ICBT are viewed as valuable by PSP. Given that these characteristics align with constructs on the CFIR that facilitate successful implementation, these characteristics should be included when designing and delivering ICBT to PSP (e.g., tailoring ICBT to PSP, free service, no referral needed). Finally, the findings provide insight into how PSP leaders would like ICBT funded, indicating that there is a need to seek out government grants or funding rather than funding through private services or passing the costs onto PSP or PSP organizations. Although this study was conducted in Canada, the results may have implications for those interested in providing tailored ICBT to PSP or other groups around the world. As an example of how implementation research can be applied to facilitate program sustainment, this study may also help guide the implementation research efforts of other groups involved in ICBT, PSP mental health, or other areas.

### 4.5. Limitations

A key limitation of this research is that participants self-selected whether or not to take part in the study after being contacted by the PSPNET team. Therefore, the results could be impacted by a self-selection bias, such that those who decided to participate may have been more attuned to the need for mental health services for PSP and therefore more supportive of PSPNET. The current study also employs numerous statistical tests comparing PSP leaders and non-leaders and those with and without prior knowledge of PSPNET, which increases the family-wise risk of errors. The current study is exploratory and was designed to provide a broad overview of Canadian PSP’s perspectives on ICBT and factors that may influence them; findings should be considered preliminary pending future replication.

## 5. Conclusions

ICBT has been tailored to meet the needs of PSP but now needs to be sustained and expanded to be of the greatest benefit. The current study sought to identify perceptions of ICBT for PSP more broadly in Canada than previously studied and explored any differences in perceptions among those with or without prior knowledge of ICBT and among leaders and non-leaders. Furthermore, there are facilitators and barriers to expanding and sustaining ICBT for PSP. PSP from diverse sectors across Canada reported perceiving ICBT as beneficial and reported a number of advantages that set ICBT apart from other psychological services that are available to PSP. The results of the present study indicate that there is a national need for ICBT for PSP, both at the general member and leadership levels of many Canadian PSP organizations. Knowledge of ICBT for PSP appeared to be associated with more positive perceptions of ICBT, suggesting that increasing education and awareness of ICBT can facilitate positive perceptions of ICBT, which can aid in the implementation and sustainability of services. The results also suggest that PSP believe there is a need for increased and sustained funding to support a broader and ongoing availability of ICBT for PSP across Canada, with an emphasis placed on the government’s responsibility for covering this service. Factors that may facilitate this funding include increased awareness of the effectiveness of ICBT and the need for such services, which can help overcome barriers to funding (e.g., the government prioritizing other issues and a lack of political will).

## Figures and Tables

**Table 1:** Participant characteristics.

Characteristics	Total Sample (*n* = 524)
Gender, *n* (%)	
Women	239 (45.6)
Men	273 (52.1)
Non-binary	2 (0.4)
Missing data	10 (1.9)
Province, *n* (%)	
Ontario	104 (19.8)
British Columbia	104 (19.8)
Quebec	103 (19.7)
Saskatchewan	77 (14.7)
Alberta	69 (13.2)
Atlantic Canada ^a^	45 (8.5)
Manitoba	23 (4.2)
PSP Sector, *n* (%)	
Police	131 (25.0)
Correctional worker	121 (23.1)
Paramedic	92 (17.6)
Fire	66 (12.6)
Public safety communicator	50 (9.5)
Not listed (e.g., support staff)	64 (12.2)
Ethnicity, *n* (%)	
White	448 (85.5)
Indigenous (i.e., First Nations, Inuit, Metis)	21 (4.0)
Ethnic minority ^b^	35 (6.7)
Prefer not to answer	10 (1.9)
Missing data	8 (1.5)
Leadership status, *n* (%)	
Manager	144 (27.5)
Union or association representative	50 (9.5)
Not leadership	262 (50.0)
Other	68 (13.0)
Missing data	10 (1.8)
Knowledge of PSPNET, *n* (%)	
Never heard of PSPNET	377 (71.9)
Heard of PSPNET	147 (28.9)
Received services from PSPNET	16 (3.1)
Age, *n* (%)	
19–29	51 (9.9)
30–39	146 (28.2)
40–49	162 (31.3)
50+	158 (30.6)
Missing data	7 (1.3)

^a^ “Atlantic Canada” refers to the provinces of New Brunswick, Nova Scotia, and Prince Edward Island. ^b^ “Ethnic minority” refers to participants who self-identified as Asian, Black, Middle Eastern, or South Asian.

**Table 2:** Perceptions of need for mental health and ICBT.

	Heard of PSPNET		Self-Identified Leader	
	Yes (*n* = 147)	No (*n* = 376)	Yes (*n* = 262)	No (*n* = 262)
Attitudes, *M (SD)*				
Mental health problems are common in my occupation	4.48 (0.67)	4.53 (0.71)	4.3 (0.65)	4.51 (0.71)
People in my occupation do not receive the mental health support they need	4.16 (0.95)	4.22 (0.89)	4.18 (0.87)	4.23 (0.93)
PSPNET could help people in my occupation improve their mental health	4.37 (0.72)	4.17 (0.72) **	4.27 (0.72)	4.19 (0.73)
PSPNET should be easily and freely accessible to people in my occupation	4.68 (0.61)	4.56 (0.59) *	4.61 (0.58)	4.58 (0.62)
As a PSP, I have experienced symptoms of depression, anxiety, or posttraumatic stress injuries	4.08 (1.15)	4.20 (1.02)	4.16 (1.08)	4.18 (1.04)
I think I could benefit (or previously could have benefitted) from PSPNET	4.07 (0.99)	4.08 (0.85)	4.03 (0.89)	4.12 (0.89)
I have experienced challenges accessing professional help for symptoms of depression, anxiety, or posttraumatic stress injuries	3.25 (1.21)	3.55 (1.17) **	3.44 (1.18)	3.48 (1.19)
If I needed help, I would prefer to use some other service	2.54 (1.04)	2.86 (0.92) ***	2.69 (0.98)	2.84 (0.94)

* *p* < 0.05, ** *p* < 0.01, *** *p* < 0.001; Participants provided responses on a 5-point Likert-type scale ranging from 1 (strongly disagree) to 5 (strongly agree). Some participants did not respond to all items; the actual number of responses summarized in each row of this table ranges from 520 to 524, with one exception. Namely, the item “If I needed help, I would prefer to use some other service”, had 368 responses from participants who had heard of PSPNET and 142 responses from participants who had not. It had 258 responses from participants who identified as leaders and 252 responses from participants who did not.

**Table 3:** Perceived advantages of ICBT.

Advantages	Heard of PSPNET		Self-Identified Leader	
	Yes (*n* = 147)	No (*n* = 377)	Yes (*n* = 262)	No (*n* = 262)
Accessible, *M* (*SD*)				
Accessible at any time and location	3.81 (0.53)	3.71 (0.60)	3.77 (0.52)	3.70 (0.64)
Minimal wait time	3.80 (0.52)	3.74 (0.54)	3.80 (0.47)	3.72 (0.59)
Website easy to use	3.50 (0.73)	3.45 (0.71)	3.54 (0.69)	3.39 (0.74) *
No referral needed	3.68 (0.72)	3.65 (0.65)	3.67 (0.66)	3.65 (0.68)
Free service	3.79 (0.61)	3.74 (0.64)	3.73 (0.65)	3.78 (0.61)
Therapist-Guided, *M* (*SD*)				
Initial online and telephone assessment	3.48 (0.77)	3.33 (0.82) *	3.42 (0.76)	3.33 (0.86)
Referral to other services if needed	3.61 (0.68)	3.46 (0.74) *	3.53 (0.67)	3.47 (0.78)
Therapist guidance for 16 weeks	3.76 (0.59)	3.59 (0.68) **	3.68 (0.59)	3.60 (0.72)
Flexibility of therapist contact (email/phone)	3.71 (0.64)	3.59 (0.66)	3.65 (0.62)	3.60 (0.70)
Customized for PSP, *M* (*SD*)				
Stories and examples from PSP	3.34 (0.85)	3.14 (0.82) *	3.25 (0.82)	3.15 (0.83)
Developed with input from PSP	3.50 (0.74)	3.36 (0.76)	3.44 (0.74)	3.37 (0.77)
Tailored for PSP	3.64 (0.66)	3.48 (0.72) *	3.54 (0.69)	3.50 (0.73)
Effective Format and Resources, *M* (*SD*)				
Additional resources	3.58 (0.68)	3.48 (0.70)	3.51 (0.69)	3.50 (0.69)
Effectiveness through peer-review	3.51 (0.74)	3.42 (0.73)	3.45 (0.74)	3.44 (0.73)
Exercises to help build skills	3.53 (0.71)	3.45 (0.71)	3.51 (0.68)	3.44 (0.73)
Online delivery	3.58 (0.70)	3.40 (0.77) **	3.53 (0.71)	3.37 (0.79) **
Material can be downloaded	3.66 (0.69)	3.52 (0.71)	3.58 (0.67)	3.55 (0.75)
Other, *M* (*SD*)				
Greater privacy than other treatments	3.54 (0.77)	3.36 (0.86) *	3.45 (0.80)	3.36 (0.88)
Extensively Researched	3.60 (0.72)	3.49 (0.76)	3.55 (0.74)	3.49 (0.75)
Complements existing services	3.54 (0.72)	3.36 (0.74) **	3.43 (0.70)	3.38 (0.78)

* *p* < 0.05, ** *p* < 0.01. Ratings were made on a 4-point Likert scale ranging from 1 (not an advantage) to 4 (major advantage). Some participants did not respond to all items; the actual number of responses summarized in each row of this table ranges from 520 to 524.

**Table 4:** Perceived concerns about ICBT.

	Heard of PSPNET		Self-Identified Leader	
	Yes (*n* = 147)	No (*n* = 376)	Yes (*n* = 262)	No (*n* = 262)
Concerns, *M (SD)*				
Absence of face-to-face interactions (i.e., therapist cannot observe non-verbal cues)	2.58 (0.88)	2.66 (0.92)	2.67 (0.87)	2.60 (0.95)
Requires internet access and some comfort with technology to start service and download materials	1.99 (0.96)	1.96 (0.93)	1.98 (0.94)	1.96 (0.93)
Confidentiality of information	2.10 (1.11)	2.34 (1.04) *	2.24 (1.10)	2.30 (1.03)
Reading material could be fatiguing	2.26 (0.93)	2.32 (0.90)	2.35 (0.90)	2.25 (0.92)
Treatment requires at least an 8-week time commitment	2.05 (0.90)	1.98 (0.86)	2.06 (0.91)	1.94 (0.84)
Treatment material may not meet my individual needs	2.31 (0.85)	2.42 (0.85)	2.36 (0.82)	2.42 (0.87)
Treatment should be completed within 16 weeks	2.18 (0.89)	2.18 (0.94)	2.20 (0.91)	2.17 (0.91)
ICBT may require more motivation than face-to-face therapy	2.40 (0.90)	2.45 (0.94)	2.43 (0.92)	2.44 (0.94)

* *p* < 0.05; Participants rated their responses on a 4-point Likert-type scale ranging from 1 (not a concern) to 4 (major concern). Some participants did not respond to all items; the actual number of responses summarized in each row of this table ranges from 520 to 524.

**Table 5:** Leaders’ perceptions of need for mental health services and support of ICBT services.

	Heard of PSPNET	
	Yes *(n* = 96)	No *(n* = 159)
Attitudes, *M (SD)*		
Addressing mental health problems is a strategic priority with the specific organization in which I work	3.76 (1.16)	3.69 (1.16)
There is pressure from within my organization to improve mental health	3.47 (1.06)	3.55 (1.06)
There is pressure from outside my organization to improve mental health	3.59 (1.08)	3.64 (1.01)
I believe having PSPNET available to people in my organization should be a priority to my organization	4.38 (0.74)	4.09 (0.85) **
I believe PSPNET would be effective for improving the mental health of members of my organization	4.34 (0.78)	4.08 (0.84) *
If PSPNET were available to people in my organization, my organization would be able to easily promote PSPNET so PSP would use it	4.23 (0.75)	3.99 (0.87) *
I believe people in my organization would use PSPNET if it were available	4.05 (0.80)	3.88 (0.85)
PSPNET complements other mental health services available to PSP in my organization	4.03 (0.81)	3.99 (0.81)
I believe PSPNET would be an effective treatment option for PSP in my organization	3.96 (1.01)	3.91 (0.88)
I believe people in my organization would like to prioritize a different mental health program rather than PSPNET	3.11 (1.18)	3.10 (0.90)
I would personally advocate for people in my organization to have access to PSPNET	4.36 (0.84)	3.93 (0.91) ***

* *p* < 0.05, ** *p* < 0.01, *** *p* < 0.001; Participants rated their responses on a 5-point Likert-type scale ranging from 1 (strongly disagree) to 5 (strongly agree). Some participants did not respond to all items; the actual number of responses summarized in each row of this table ranges from 252 to 255, with two exceptions. Namely, the item “I would personally advocate for people in my organization to have access to PSPNET” had 74 responses from leaders who had heard of PSPNET and 133 responses from leaders who had not. Likewise, the item “I believe people in my organization would like to prioritize a different organization” had 144 responses from leaders who had heard of PSPNET and 91 responses from leaders who had not.

**Table 6:** Perceptions of how to best fund PSPNET (*n* = 153).

Categories	Description	References, *n* (%)
Government funding	PSPNET should be funded through government funding, be it provincial or federal	123 (80.3)
Employer funding	PSPNET should be funded through PSP employers (e.g., Worker’s Compensation Board contributions, employee, and family assistance programs)	18 (11.8)
Unsure	Participants reported PSPNET should be funded, but they were not sure where the funds should come from	6 (3.9)
Charitable donations	PSPNET should be funded by charitable donations from non-profit organizations	4 (2.6)
Payroll deduction	PSNET should be funded by PSP themselves through payroll deduction	2 (1.3)

**Table 7:** Perceptions of factors that will facilitate funding of PSPNET (*n* = 101).

Categories	Description	References, *n* (%)
Evidence of success	PSPNET would be able to facilitate more funding if they present evidence of the success of ICBT services	38 (37.6)
Raising awareness of PSP mental health needs	PSPNET would be able to facilitate more funding by advocating for mental health among PSP, raising awareness of PSP mental health needs in public forums, and promoting and advertising PSPNET’s free ICBT services	23 (22.8)
Endorsements from trade unions	PSPNET would be able to facilitate funding by obtaining support from trade unions	12 (11.9)
Lobbying the government	PSPNET would be able to facilitate funding by lobbying the government	11 (10.9)
Other	Participants reported PSPNET should be funded, but they were not sure how this should be achieved	8 (7.9)
Endorsements from government health agencies	PSPNET would be able to facilitate funding by obtaining support from government health agencies (e.g., Public Health Agency of Canada)	6 (5.9)

**Table 8:** Perceptions of barriers for obtaining more funding for PSPNET (*n* = 123).

Categories	Description	References, *n* (%)
Competing priorities	The government prioritizes other issues ahead of funding PSP mental health	26 (21.1)
Lack of political will	There is a lack of understanding of the work PSP do, driving political ambivalence	22 (17.9)
Lack of funding	Lack of resources or limited budgets will impede PSPNET funding	20 (16.3)
Unsure	Participants indicated they were unsure	16 (13.0)
Poor results and low participation	Funding would be impacted if there were poor results or low participation	16 (13.0)
Unrecognized need	PSP do not understand the importance of ICBT services or recognize a need for it	13 (10.6)
Negative public attitudes	Belief that the general public are not concerned with PSP mental health	10 (8.1)
Lack of promotion	PSP are unaware of services	8 (6.5)

## Data Availability

As participant consent for widespread data sharing was not obtained, the data in this study will not be made available.

## Supplementary Materials

The following supporting information can be downloaded at: https://www.mdpi.com/article/10.3390/ijerph20085592/s1, Document S1: PSPNET Informational Video Script; Table S1: PSPNET survey items organized by the Innovation Domain of the Consolidated Framework for Implementation Research.
