# Supplementary material for: Sustaining and Expanding Internet-Delivered Cognitive Behavioral Therapy (ICBT) for Public Safety Personnel across Canada: A Survey of Stakeholder Perspectives
Source: Int J Environ Res Public Health. 2023 Apr 20;20(8):5592. doi: 10.3390/ijerph20085592 (PMC10139003; doi:10.3390/ijerph20085592)
Supplement: Supplementary file 1 [file ijerph-20-05592-s001.zip › ijerph-2212318-supplementary.pdf]

Supplementary Document Material 1: PSPNET Informational Video Script and Brochure

PSP #1 TF- I start to get a little light headed, almost dizzy at times.

PSP #2 - Depression, anxiety, nightmares is the big one.

PSP #3 - You feel weird but you don't know why you feel weird.

PSP #2 - Your heart is racing so bad that you can't get back to sleep for hours.

PSP #1 - I even ended up going to the hospital one time because I was worried I was having a heart attack.

PSP #4 - It can sometimes be mentally triggering or emotionally triggering.

PSP #2 - The nightmares come and go, I usually have two or three a week.

PSP #1 - If I do survive am I going to be able to be a firefighter?

PSP #3 - I was angry all the time, I didn't even want to be around myself.

PSP #2 - It is almost like it's a dreary day, day in and day out.

PSP #1 - And it seems like sometimes we are fighting a losing battle.

Onscreen text - *PSPNET is a clinical unit located at the University of Regina.*

The unit is led by Dr. Heather Hadjistavropoulos, one of Canada's leading scholars on Internet-delivered Cognitive Behaviour Therapy (ICBT).

In partnership with the Canadian Institute for Public Safety Research and Treatment, PSPNET provides treatment and conducts research on various aspects of ICBT for Public Safety Personnel.

The aim of PSPNET's ICBT program is to assist Public Safety Personnel in improving their mental health and wellbeing.

PSPNET Clinical Research Associate - As a psychologist working with PSPNET I get to witness firsthand the benefits and value of online therapy. We have tailored our program to meet the unique needs of public safety personnel and first responders. This information was based on detailed interviews with over 125 public safety personnel in Saskatchewan and Quebec. Once a participant has completed our online screening process and we determine that online therapy is the most appropriate support for them, they are enrolled in one of our two courses. These courses are typically eight weeks long and are offered with optional weekly therapist support for up to 16 weeks. The courses include five main lessons which are presented in an engaging manner and include tools and strategies intended to help manage them their symptoms in a healthier way. Clients who have completed the course have provided great feedback on the program. Public safety personnel have shared that they found the content relatable and were really pleased the courses were flexible so they could work through the lesson material at their own pace.

Onscreen text - *Based on feedback from public safety personnel who accessed and completed our program:*

- *97% BELIEVE IT WAS WORTH THEIR TIME*

- *96% WOULD REFER A FRIEND*
- *88% REPORTED INCREASED CONFIDENCE IN MANAGING SYMPTOMS*

Based on feedback from Public Safety Personnel who accessed and completed our program, 89% have reduced clinical symptoms in areas such as depression, anxiety, and posttraumatic stress.

PSPNET Clinical Research Associate - It is so encouraging to see public safety personnel reaching out to us and finally getting the support they need and deserve for their mental health and wellbeing. I think we are now just beginning to see how impactful this innovative mental health platform can be for improving access to support services. But more importantly, public safety personnel are always there when we need them and I am just so happy that we are finally able to return the favor through the programs we offer at PSPNET.

PSP #1 - It is really nice to see that this issue is getting the attention that it needs because there are first responders out there that are suffering in silence.

PSP #2 - It feels like people care and for first responders that is genuinely comforting.

PSP #4 - It was actually very inspiring to see firsthand all the research and resources put into this project.

PSP #3 - It really gives me peace of mind knowing that I can talk to somebody about how I'm feeling and that everything I say will stay confidential. It feels good knowing that I am not alone.

Onscreen text - *Public Safety Personnel need many important tools. We provide one more.*

PSPNET Courses are free, flexible, confidential, and do not require a referral.

PSPNET is available to current, former, and volunteer Public Safety Personnel from many sectors, such as

- *BORDER SERVICES OFFICERS*
- *PUBLIC SAFETY COMMUNICATORS (e.g., 911, dispatch)*
- *CORRECTIONAL WORKERS*
- *FIREFIGHTERS*
- *PARAMEDICS*
- *POLICE*

In 2020 and 2021, PSPNET has provided ICBT to over 300 Public Safety Personnel in Saskatchewan and Quebec.

We hope to extend our services to PSP in other provinces soon.

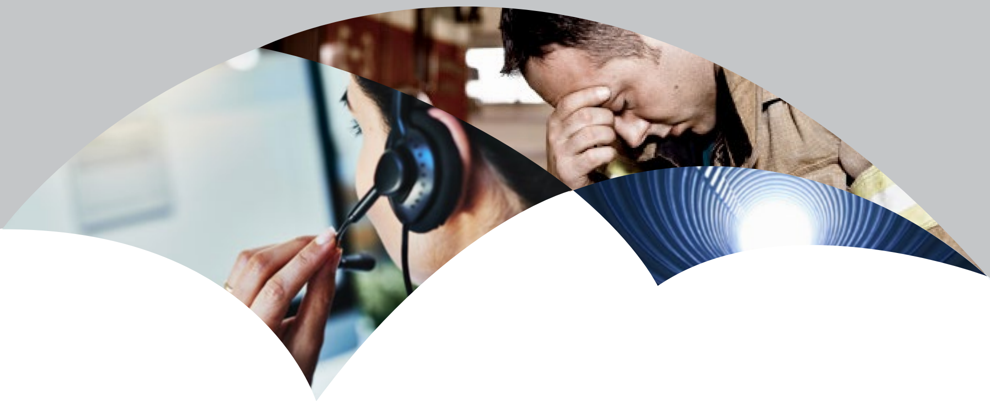

PSPNET

Improving the Wellbeing of Public Safety Personnel Through Internet-Delivered Cognitive Behaviour Therapy:

An Accessible, Online, Confidential, Evidence-Based Treatment

What is PSPNET ?

PSPNET is a team of clinicians and researchers based at the University of Regina who are delivering and evaluating Internet-delivered cognitive behaviour therapy (ICBT) for Public Safety Personnel (PSP). The team, led by Dr. Heather Hadjistavropoulos, one of Canada's leading scholars on ICBT, worked with PSP to tailor ICBT to meet PSP's unique needs.

Who We Serve

Current and former PSP including, but not limited to:

- Border services officers
- Public safety communicators
- Correctional workers
- Firefighters (career and volunteer)
- Indigenous emergency managers
- Operational intelligence personnel
- Paramedics
- Police (municipal, provincial, federal)
- Search and rescue personnel

PSPNET is now accepting clients in Saskatchewan and Quebec.

In order to participate in PSPNET, PSP must:

- Be 18 years of age or older
- Have access to a computer with internet
- Be willing to provide an emergency medical contact
- Be willing to periodically answer questionnaires to help PSPNET evaluate its services

PSP reporting severe problems with alcohol, drugs, mania, or psychosis, or at high risk of suicide, are referred to face-to-face services.

How We Help

- Online modules supplemented with therapist support, one to two times per week as needed through secure e-mails or phone calls for 8-16 weeks depending on need
- Addresses anxiety, depression, and posttraumatic stress
- Includes 5 lessons and additional resources to help manage other concerns (e.g., anger, sleep, communication)
- Helps clients understand and cope with patterns of thoughts, behaviours, and feelings that cause distress or lead to difficulties in everyday functioning
- PSPNET is free and easily accesible for clients who report barriers to care (e.g., geographic, logistical, limited resources, stigma barriers)
- PSPNET follows strict ethical and legal guidelines to protect client confidentiality

What Our Research Shows

Initial outcomes of PSPNET are promising. Out of the clients who have completed the course:

- 89% experienced reduced clinical symptoms in areas such as depression, anxiety, and posttraumatic stress
- 88% reported increased confidence in managing symptoms
- 97% believe it was worth their time
- 96% would refer a friend

This study has been approved by the University of Regina Research Ethics Board.

www.pspnet.ca

Email: pspnet@uregina.ca | Phone: (306) 337-7233 (SAFE) | Toll free number: 1-833-317-7233 (SAFE)

Funded by the Government of Canada | Canada

CIPSRT Canadian Institute for Public Safety Research and Treatment

ICRTSP Institut canadien de recherche et de traitement en sécurité publique

University of Regina

Supplementary Table S1: PSPNET survey items organized by the *Innovation Domain* of the Consolidated Framework for Implementation Research.

CFIR Construct	CFIR Definition	Advantages*	Concerns*
Innovation Source	The group that developed and/or visibly sponsored use of the innovation is reputable, credible, and/or trustable.	Developed with input from PSP	
Innovation Evidence-Base	The innovation has robust evidence supporting its effectiveness.	Extensively Researched<br>Effectiveness through peer-review	
Innovation Relative Advantage	The innovation is better than other available innovations or current practice.	Accessible at any time and location<br>Minimal wait time<br>No referral needed<br>Greater privacy than other treatments	Absence of face-to-face interactions (i.e., therapist cannot observe non-verbal cues)
Innovation Adaptability	The innovation can be modified, tailored, or refined to fit local context or needs.	Stories and examples from PSP<br>Tailored for PSP<br>Compliments existing services	Treatment material may not meet my individual needs
Innovation Trialability	The innovation can be tested or piloted on a small scale and undone.	Referral to other services if needed	
Innovation Complexity	The innovation is complicated, which may be reflected by its scope and/or the nature and number of connections and steps.	Website easy to use	Requires internet access and some comfort with technology to start service and download materials<br>ICBT may require more motivation than face-to-face therapy
Innovation Design	The innovation is well designed and packaged, including how it is assembled, bundled, and presented.	Initial online and telephone assessment<br>Therapist guidance for 16 weeks<br>Flexibility of therapist contact (email/phone)<br>Additional resources<br>Exercises to help build skills<br>Online delivery<br>Material can be downloaded	Confidentiality of information<br>Reading material could be fatiguing<br>Treatment requires at least an 8-week time commitment<br>Treatment should be completed within 16 weeks
Innovation Cost	The innovation purchase and operating costs are affordable.	Free service	

Note: Constructs and definitions of constructs in this table are taken directly from the updated Consolidated Framework for Implementation Research (Damschroder et al., 2022).
*Ratings for advantages were made on a 4-point Likert scale ranging from 1 (Not an advantage) to 4 (Major advantage). Ratings for concerns were made on a 4-point Likert scale ranging from 1 (Not an concern) to 4 (Major concern)
